# A Plant Virus Manipulates the Behavior of Its Whitefly Vector to Enhance Its Transmission Efficiency and Spread

**DOI:** 10.1371/journal.pone.0061543

**Published:** 2013-04-16

**Authors:** Ana Moreno-Delafuente, Elisa Garzo, Aranzazu Moreno, Alberto Fereres

**Affiliations:** Instituto de Ciencias Agrarias (ICA), Consejo Superior de Investigaciones Científicas (CSIC), Madrid, Spain; Volcani Center, Israel

## Abstract

Plant viruses can produce direct and plant-mediated indirect effects on their insect vectors, modifying their life cycle, fitness and behavior. Viruses may benefit from such changes leading to enhanced transmission efficiency and spread. In our study, female adults of *Bemisia tabaci* were subjected to an acquisition access period of 72 h in *Tomato yellow leaf curl virus* (TYLCV)-infected and non-infected tomato plants to obtain viruliferous and non-viruliferous whiteflies, respectively. Insects that were exposed to virus-infected plants were checked by PCR to verify their viruliferous status. Results of the Ethovision video tracking bioassays indicated that TYLCV induced an arrestant behavior of *B. tabaci*, as viruliferous whitefly adults remained motionless for more time and moved slower than non-viruliferous whiteflies after their first contact with eggplant leaf discs. In fact, Electrical Penetration Graphs showed that TYLCV-viruliferous *B. tabaci* fed more often from phloem sieve elements and made a larger number of phloem contacts (increased number of E1, E2 and sustained E2 per insect, p<0.05) in eggplants than non-viruliferous whiteflies. Furthermore, the duration of the salivation phase in phloem sieve elements (E1) preceding sustained sap ingestion was longer in viruliferous than in non-viruliferous whiteflies (p<0.05). This particular probing behavior is known to significantly enhance the inoculation efficiency of TYLCV by *B. tabaci*. Our results show evidence that TYLCV directly manipulates the settling, probing and feeding behavior of its vector *B. tabaci* in a way that enhances virus transmission efficiency and spread. Furthermore, TYLCV-*B. tabaci* interactions are mutually beneficial to both the virus and its vector because *B. tabaci* feeds more efficiently after acquisition of TYLCV. This outcome has clear implications in the epidemiology and management of the TYLCV-*B. tabaci* complex.

## Introduction

Animal viruses can often exit and enter hosts directly through cell membranes and can easily spread from host to host as animals can move by their own transporting viruses and other pathogens from place to place. Plant viruses, however, often rely on arthropod vectors to cross the cell wall boundary and move from plant to plant and over distant regions [1]. The consequence is that arthropod vector behavior has strong ecological and evolutionary implications for the plant viruses they transmit. Viruses as well as other parasites may evolve in a manner that selective advantages may appear increasing their transmission rate and their ability to spread from host to host. This has been reported with several animal infecting parasites that induce increased biting rates in their arthropod vectors such as mosquitoes infected with La Crosse encephalitis virus [2] and Dengue-2 virus [3].

Most plant viruses are transmitted by insect vectors, thus depend on their behavior, transmission and dispersal capacity to move from plant to plant and to spread to distantly-located regions [4]. Hemipterans, which include aphids, whiteflies and hoppers, involve most of the phytopathogenic virus vectors [5]. Transmission of plant viruses is mediated by the piercing-sucking mouthparts of these insects, named stylets, when penetrate through the intercellular spaces and establish feeding sites in phloem sieve elements [6]. Sweet potato whitefly, *Bemisia tabaci* Gennadius (Hemiptera: Aleyrodidae) is a destructive pest of horticultural crops and ornamental plants worldwide [7], [8], especially because of its role as a vector of plant viruses [9], [10]. Actually, *B. tabaci* is a species complex containing at least 28 morphologically indistinguishable species [11], [12]. Among these, the ‘Mediterranean’ putative species [12], [13] has been previously referred as the Q biotype and is the most extended in Spain. However, this recent phylogenetic delineation has not been officially recognized [14], and the term Q biotype of *B. tabaci* is still commonly used to refer to the ‘Mediterranean’ putative species.

*Tomato yellow leaf curl virus* (TYLCV), a member of the Begomovirus (family Geminiviridae), affects tomato cultures in many tropical and subtropical regions [15]. TYLCV is transmitted in a persistent, circulative manner by *B. tabaci* [16]. Begomoviruses and whiteflies have developed co-evolutionary mechanisms that ensure (i) the survival and the efficient transmission of the virus, and (ii) the safeguard of the insect host from possible deleterious effects of the virus [17]. The specific virus-vector interactions that determine begomovirus transmission are complex, involving not only the virus and vector but also the host plant and environment. In addition to the nature of virus acquisition and association with the vector, vector landing and probing on the plant source as well as vector feeding patterns may influence the efficiency of virus transmission [4], [10]. Therefore, changes in the host plant that alter vector behavior could change transmission and disease dynamics [18].

Plant viruses may modify the behavior of their vectors in different ways. Relatively little research has explored solely the direct effects of plant pathogens on their vectors when they are ingested and circulate through their body. In fact, most of the studies have concentrated on the indirect effects mediated by the plant when a virus induces changes in vector performance and behavior or in the preference for its plant host after infection [19]–[23], or the mixed effects (direct plus indirect effects) [24], [25]. Evaluating the indirect effects, from the pathogen stand point, the virus will benefit when the attractiveness of the infected host plant is maximum until the vector lands and probes on the plant. Then, after virus acquisition, the residence time of vectors on infected plants should be reduced to enhance virus movement to neighboring healthy plants and therefore, increase the rate of spread [4]. This hypothesis has been confirmed in specific virus-vector relationships [26] as well as for the plant-pathogenic bacteria *Candidatus* Liberibacter asiaticus, which alters the host preference behavior of its psyllid vector *Diaphorina citri* increasing its attractiveness to infected plants [27]. From the vector stand point, the preference for infected plants may have beneficial or detrimental effects for its performance. Mutualism relationships between plant viruses and their insect vectors have been observed in some Hemipterans. In the case of *B. tabaci* biotype B and *Tomato yellow leaf curl China virus* (TYLCCNV) interactions, Jiu et al. [20] found that fecundity, longevity and population density of whiteflies increased when feeding on virus-infected tobacco plants. Zhang et al. [22] explained that this mutualism is a consequence of changes in plant defense responses after begomovirus infection. In fact, the infection by TYLCCNV interferes with the synthesis of jasmonic acid, which is used by plants against herbivory attack, resulting in enhanced performance of *B. tabaci.* Furthermore, Luan et al. [23] added to this research that terpenoids, important plant defensive compounds, were depleted by begomovirus infection, favoring whitefly fitness. Another virus-vector mutualism relationship was found when the aphid *Sitobion avenae,* fed on *Barley yellow dwarf virus* (BYDV) infected wheat [28]. Aphids had a significantly shorter developmental time, a greater fecundity, and a greater intrinsic rate of natural increase on BYDV-infected than on non-infected plants. Conversely, the infection of soybean with alfalfa mosaic, soybean mosaic and bean pod mottle viruses, inoculated separately, had several negative effects in the aphid *Aphis glycines* performance, decreasing the aphid population growth rate and the aphid density on infected plants [29].

Nevertheless, plant viruses may also interact directly with their insect vectors, altering their behavior to enhance their own spread. Recently, Stafford et al. [30] found that *Tomato spotted wilt virus* (TSWV) directly modifies the feeding behavior of its vector *Frankliniella occidentalis* increasing its ability to transmit the virus. Ingwell et al. [31] found that the settling behavior of the aphid *Rhopalosiphum padi* changed after acquisition of BYDV. Aphids that acquired the virus preferred to feed on non-infected wheat plants, while non-infective aphids preferred BYDV-infected plants. Some studies have suggested that TYLCV, as other geminivirus, has some pathogenic characteristics and could be deleterious to its vector, *B. tabaci*. In fact, the presence of TYLCVs in *B. tabaci* has been associated with a decrease in the insect longevity and fertility [20], [32], [33]. Furthermore, a recent study demonstrated an imbalanced nutrition of whiteflies infected with TYLCCNV [25]. However, limited studies of changes in the behavior of whiteflies due to infection with plant viruses such as TYLCVs have been reported.

In the present work we studied the settling and feeding behavior of *B. tabaci* after exposure to TYLCV using eggplants, a preferred host of the vector but immune to the virus. The study was conducted using the automatic video tracking system Ethovision [34] to compare the settling behavior of viruliferous and non-viruliferous whiteflies. The electrical penetration graph (EPG) technique [35] was used to study the probing and feeding behavior of *B. tabaci* using a similar approach as previously described [36], [37]. It is known that the transmission efficacy of TYLCV by *B. tabaci* depends on specific activities such as the time spent on salivation into the phloem sieve elements [38]. Because TYLCV transmission and spread depends on the feeding behavior of its vector *B. tabaci*, we hypothesized that the virus could have co-evolved to alter the behavior of the vector to enhance its own spread.

## Materials and Methods

### Ethics Statement

The colony was initiated from field-collected individuals from Málaga, Spain and maintained by serial transfer at ICA-CSIC, Madrid, Spain. No specific permits were required for the described studies. No specific permissions were required for the locations/activities described in the manuscript. Location is not a private property.

### Whitefly Population, Virus Isolate and Plants

A colony of biotype Q of *B. tabaci*, also referred in some recent reports as the ‘Mediterranean’ putative species [12], was reared on potted eggplants (*Solanum melongena* L. cv. Black Beauty) within metal-frame cages covered by insect-proof net in a greenhouse (25∶21°C day:night temperature, 80% RH). The colony was initiated from field-collected individuals from Málaga, Spain and maintained by serial transfer at ICA-CSIC, Madrid, Spain. Identity of biotype status of the population in rearing was periodically confirmed by determining the sequence of cytochrome oxidase I mitochondrial gene according to the protocol of Frolich et al. [39] and performing a BLAST procedure with one reference sequence of biotype Q from Spain in the Gen Bank: AF342769 (data not shown).

TYLCV isolate used was a TYLCV-IL type [ES:Alm:Pep:99] with GenBank accession number AJ489258, described by Morilla et al. [40]. The virus isolate was maintained in tomato plants (*Lycopersicon esculentum* L. cv. Marmande) and maintained by whitefly-mediated transmission. Tomato plants were infected with TYLCV one month before conducting the experiments.

Eggplants were sown in cell trays and transplanted at the two-leaf stage to individual pots containing vermiculite and soil substrate (1∶1 v/v) and grown in a chamber at a 24∶20°C day:night temperature, 80% RH and 16∶8 h light:dark photoperiod. Eggplants were used because they are excellent host plants for *B. tabaci*, but immune to TYLCV, excluding any plant-mediated indirect modifications due to virus infection that could mask the direct effects of the virus on its vector.

No specific permits were required for the described studies.

### Generation of TYLCV-infected Plants

Clip cages with 50 adults of *B. tabaci* were placed in young terminal leaflets of tomato plants showing characteristic TYLCV symptoms for an acquisition access period (AAP) of 72 h. Then, clip cages were open and whiteflies were released on uninfected 2-3-leaf stage tomato test plants for an additional 48 h of inoculation access period. Whiteflies were then removed from tomato plants with a handmade vacuum aspirator and plants were placed in insect-proof cages in a greenhouse (same conditions as described above).

### Generation of Viruliferous and Non-viruliferous *B. tabaci* Colonies

Female whitefly adults of a wide range of age (from 1 to 15 days old) were collected from rearing cages of the virus-free colony and mixed together to generate the viruliferous and non-viruliferous whiteflies used to conduct the experiments. Viruliferous whiteflies were obtained by confinement of virus-free *B. tabaci* adults on TYLCV-infected tomato plants (6-leaf growth stage) during a 72 h AAP. To obtain non-viruliferous whiteflies, virus-free *B. tabaci* adults were confined for 72 h in healthy tomato plants of the same growth stage.

A set of 100 *B. tabaci* individuals under each experimental group that were not used for the behavioral studies was stored at −80°C for verifying their status (TYLCV-viruliferous or non-viruliferous). Virus-infection status was determined by squash-capture PCR [41]. Single whiteflies were squashed on paper with the rounded end of an Eppendorf tube. Pieces of squashed samples were inserted into Eppendorf tubes and 100 µl of Triton X-100 0,5% [42] were added, incubated at 95°C for 10 min, vortexed and placed on ice. Two microliters of each extract were directly used for the PCR assays.

According to Ghanim et al. [43], a _∼_410–bp TYLCV DNA fragment was amplified using the two following primers V61 (nucleotides 61–80, viral strand, ATACTTGGACACCTAATGGC) and C473 (nucleotides 473–457, complementary strand, AGTCACGGGCCCTTACA). The cocktail for PCR amplification was a mixture of 50 µl containing 10 mM Tris-HCl pH 8,8, 2,5 mM MgCl_2_, 0,25 mM dNTPs, 0,4 µM of each primer (V61 and C473), 2 µl of DNA sample and 2 units of TaqDNA Polymerase (Biotools®).

The PCR amplification conditions were the same as proposed by Ghanim et al. [43]: a initial denaturation phase of 3 min at 95°C, followed by 33 cycles of amplification 94°C for 1 min, 45°C for 1 min and 72°C for 2 min; and 10 min at 72°C. As controls, non-viruliferous whiteflies were similarly managed. PCR products (8 µl) were subjected to electrophoresis in a 1.5% agarose gel, stained with ethidium bromide.

### Comparison of the Settling Behavior of TYLCV-viruliferous and Non-viruliferous *B. tabaci* Adults

Leaf discs of 2.5 cm in diameter were cut from well-developed eggplant leaves, avoiding pronounced central nerves to prevent interferences with the camera tracking system. Discs were placed showing the abaxial face on the center of a Petri dish of 12.5 cm in diameter. Each disc was fixed to the Petri dish on the middle surface of a 10% agar layer just before solidification. All leaf discs were used in a 2-h time interval after excision. TYLCV-viruliferous and non-viruliferous whiteflies were subjected to a 30 min starvation period before experiments began. Whitefly adult females were collected and kept individually, moved to a windowless temperature-controlled dark room (23±2°C,) and gradually placed on an ice bath (4°C) for 10 minutes until they were immobilized. Then, each adult whitefly was individually placed on the middle of the leaf disc and followed with a Panasonic CCTV video camera (model WV-CP500/g, Mastsushita Electric Industrial Co., Ltd., Japan) tracking system. The movement of whiteflies all over the experimental arena (a 4×3 cm rectangle containing the leaf disc) was tracked for 10 min and transferred to a PC computer as part of the Ethovision XT8 integrated video tracking system (Noldus Information Technology, Wageningen, The Netherlands). The Ethovision software automatically determines the point by point location of the individual insect within the experimental arena and automatically calculates several movement parameters derived from changes in position. The parameters analyzed for each recording were the following: (1) mean velocity (mm/s), (2) total distance moved (mm), (3) frequency in disc (n), (4) duration in disc (s), (5) duration of movement (s), (6) frequency of movement (n) (7) mean duration of movement (s), and (8) latency to first movement (s). Parameter descriptions are given in Noldus et al. [34], and algorithms and calculations are described by Noldus Information Technology [44].

Single whiteflies were used only once. Valid videos were those in which the whiteflies remained within the experimental arena during the 10-min recording time. Video recordings in which whiteflies started to fly or abandoned the experimental arena during the 10-min recording time were considered invalid for the purpose of parameter calculation. Trials of both treatments were run every day up to a total number of 18 and 21 TYLCV-viruliferous and non-viruliferous *B. tabaci* valid replicates, respectively. The number of invalid replicates under TYLCV-viruliferous and non-viruliferous treatments was 5 and 10, respectively. Data obtained for each parameter was transformed using log and square root standard transformations. Transformed data that followed a Gaussian distribution were analyzed by a Student t-test, whereas the non-parametric Mann-Whitney U-test was used when normality was not achieved. All statistical tests were conducted using the SPSS v.19 software [45] at a 0.05 significance level. A Chi-square 2×2 goodness of fit test was used to compare valid and invalid video recordings, using StatView [46] software.

### Comparison of the Probing and Feeding Behavior of TYLCV-viruliferous and Non-viruliferous *B. tabaci* Adults

An 8-channel Giga-Ohm DC-EPG device (EPG systems, Wageningen, The Netherlands) was used to monitor the probing and feeding activities of TYLCV-viruliferous and non-viruliferous *B. tabaci* adult females on eggplants during 8-h periods. To facilitate wiring, whiteflies were maintained for 10 minutes on an ice-bath and then transferred to the cover of a Petri dish that was filled with a layer of minced ice. Then, an extra thin gold wire (2 cm length, 12.5 µm in diameter) was attached to the whitefly pronotum with a small droplet of water based silver-conducting glue paint. The opposite extreme of the gold wire was glued with a droplet of paint to a thin copper wire (2 cm length) [47]. Another copper electrode (10 cm length, 2 mm in diameter) was inserted into the soil of the plant container. The acclimation period between the time of wiring and the beginning of EPG recording was approximately one hour, the same as described by Johnson and Walker [37]. Whiteflies were placed on the abaxial side of the first or second youngest leaf of 4-leaf growth stage eggplants. Each single whitefly was used only once and each eggplant was used a maximum of five times for EPG recording. Data acquisition was controlled by Stylet+ for Windows software (EPG Systems, Wageningen, The Netherlands) and data were analyzed with the same software after data conversion.

**Figure 1:**
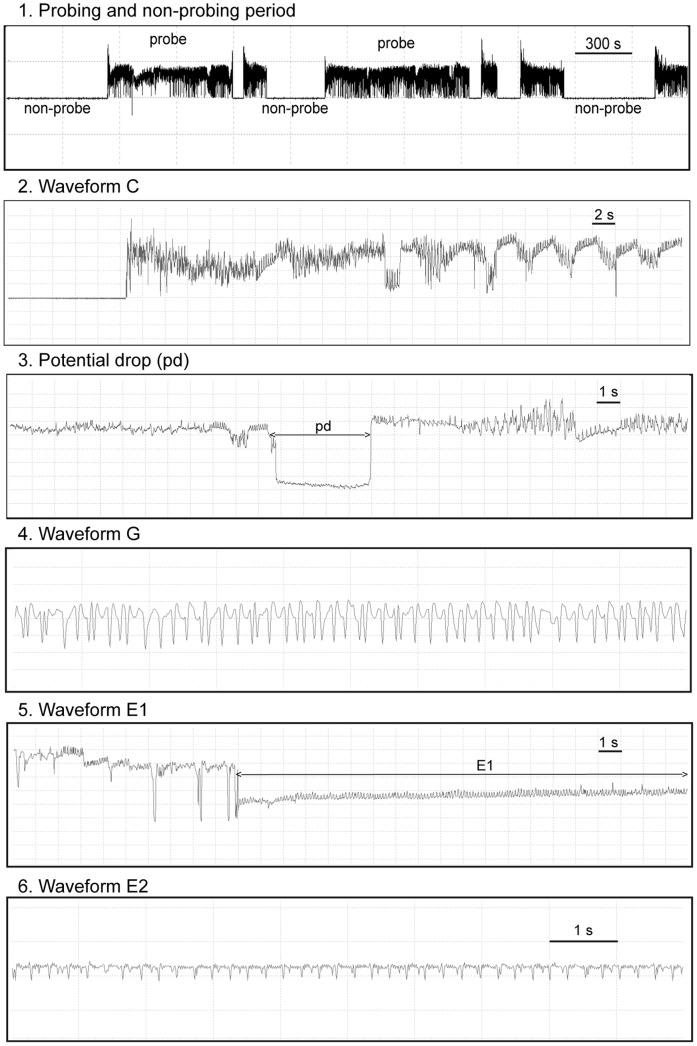
EPGs recorded for *Bemisia tabaci*. (1) Probing and non-probing period; (2) waveform C, intercellular stylet pathway; (3) waveform pd, intracellular puncture; (4) waveform G, xylem ingestion; (5) waveform E1, salivation into phloem sieve elements; (6) waveform E2, ingestion from phloem sieve elements.

Whitefly probing-associated EPG waveforms (Figure 1) were the same as previously reported by Rodríguez-Lopez et al. [48]: waveform np, non-probing behavior (no stylet contact with the leaf tissue); waveform C, intercellular apoplastic stylet pathway where the insects show a cyclic activity of mechanical stylet penetration and secretion of saliva; waveform pd (potential drops), represents brief (4–12 sec) intracellular stylet punctures during the pathway phase (C). Also, two waveforms related with the phloem activity were recorded: waveform E1, salivation into phloem sieve elements at the beginning of the phloem phase [38]; and waveform E2, correlated with passive phloem sap uptake from the sieve elements that is comparable to E2 of aphids [49]. Furthermore, waveform G was observed in EPG recordings, which represents active intake of water from xylem elements [50]. The term “probe” refers to any type of event during the period in which the stylet of an individual insect is located in contact with the plant tissue, and “no probe” refers to the event in which no contact between stylets and the plant tissue is observed (0 base line is observed) [48].

EPG sequential and non-sequential parameters related to the pathway (C and pd), phloem phase (E1 and E2) and xylem phase (G) were calculated for each EPG recording using the MS Excel workbook for automatic parameter calculation of EPG data (version 4.4.1) developed by Sarria et al. [51]. A total of 18 and 17 recordings made by non-viruliferous and viruliferous *B. tabaci* adults, respectively, were analyzed.

A total of 21 selected EPG sequential and non-sequential parameters were calculated and compared between treatments as described by Backus et al. [52]: PPW, proportion of individuals that produced a specific waveform type; NWEI, number of waveform events per insect, that is the sum of the number of events of a particular waveform divided by the total number of insects under each treatment; WDI, waveform duration (min) per insect, that is the sum of durations of each event of a particular waveform made by each individual insect that produced that waveform divided by the number of insects that performed that particular waveform under each treatment; and WDE, waveform duration (min) per event, that is the sum of the duration of the events for a particular waveform divided by the total number of events of that particular waveform under each treatment. PPW parameter was compared among the different treatment groups using a Chi-square 2×2 goodness of fit test or a Fisher’s Exact Test when the expected values were lower than 5. These tests were conducted using StatView software for Macintosh [46] and a 0.05 significance level. Transformed data of NWEI, WDI and WDE that followed a Gaussian distribution were analyzed by a Student t-test, whereas the non-parametric Mann-Whitney U-test was used for comparison when normality was not achieved. These statistical tests were conducted using the SPSS v.19 software [45] at a 0.05 significance level.

## Results

### TYLCV Detection by Squash-capture PCR

*Tomato yellow leaf curl virus* DNA was detected in all of the analyzed *B. tabaci* female adults that were subjected to the 72 h-acquisition access period on TYLCV-infected tomato plants, verifying that our virus acquisition procedure was successful. None of the whiteflies fed on non-infected plants were positive for TYLCV.

### Comparison of the Settling Behavior of TYLCV-viruliferous and Non-viruliferous *B. tabaci* Adults

The Ethovision tracking video system revealed that there was no significant differences between TYLCV-viruliferous and non-viruliferous whiteflies when we compared the number of valid (Video S1) and invalid video recordings (Video S2). Invalid video recordings were discarded for parameter calculation and statistical analysis.

Video tracking analysis (Table 1) indicated that viruliferous whitefly adults remained more time motionless on eggplant leaf discs than non-viruliferous whiteflies. The total distance travelled by whiteflies from the center-point of the eggplant disc was significantly higher in the case of non-viruliferous than viruliferous whiteflies (“Total distance moved”: t = 3.719, df = 37; p = 0.001). Furthermore, we found a higher mean velocity for non-viruliferous than for viruliferous whiteflies (“Mean velocity”: U = 78.0; p = 0.001), suggesting that TYLCV induces an arrestant response in *B. tabaci*. In fact, the duration of movement, i.e., the total time that whiteflies were moving during the 10 min duration of the recording and the frequency of the movement were significantly higher in non-viruliferous than in viruliferous whiteflies (“Duration of the movement”: U = 59.0; p = 0.000; “Frequency of the movement”: t = 2.668, df = 37; p = 0.011). No significant differences (p>0.05) were found for the rest of the parameters analyzed (Table 1). Visual inspection of the videos easily revealed the existence of a different behavior of TYLCV-viruliferous and non-viruliferous whiteflies when settling on eggplant leaf discs, suggesting that viruliferous whiteflies settled on leaf discs faster than those that were non-viruliferous. In summary, TYLCV induced an arrestant response in *B. tabaci*, as viruliferous whiteflies remained more time motionless on the disk, either resting or feeding, while non-viruliferous whiteflies were more active and traveled a longer distance and at higher speed when exposed to eggplant leaf discs.

**Table 1:** Mean (± standard error) values for parameters of movement and displacement of TYLCV-viruliferous and non-viruliferous *B. tabaci* adult females on healthy eggplants discs during ten minutes of automatic tracking.

Parameters	Whitefly		p^a^
	Non-viruliferous (n = 21)	TYLCV-viruliferous (n = 18)	
**Total distance moved (mm)**	78.16±8.21	43.36±6.39	0.001
**Duration in disc (s)**	532.01±28.31	541.32±32.61	0.282
**Frequency in disc (n)**	1.95±0.35	1.56±0.28	0.426
**Duration of movement (s)**	211.29±22.09	96.89±15.00	0.0001
**Frequency of movement (n)**	61.48±7.35	37.50±5.29	0.011
**Latency to first movement (s)**	9.98±3.28	20.16±6.03	0.294
**Mean velocity (mm/s)**	0.13±0.01	0.07±0.01	0.001

a Statistical tests performed: T-Student test on Gaussian distribution parameter “Total distance moved” and “Frequency of movement”. Mann-Whitney non-parametric test on all other variables. Significant differences (p≤0.05) between both TYLCV-viruliferous and non-viruliferous *B.tabaci*.

### Probing and Feeding Behavior of TYLCV-viruliferous and Non-viruliferous *B. tabaci* on Eggplants

The results indicated that viruliferous whiteflies reached the phloem sieve elements more often than non-viruliferous whiteflies as shown by a significantly higher number of all phloem-related activities (number of waveform events per insect, NWEI) (Table 2). The number of phloem salivation (“E1”) and ingestion (“E2” and “sustained E2”) events was almost significantly four times greater in viruliferous than in non-viruliferous whiteflies. The proportion of viruliferous whiteflies (PPW = 11/17) that produced E2 and sustained E2 was significantly higher than non-viruliferous whiteflies (PPW = 5/18). There was also a trend suggesting that viruliferous whiteflies spent longer time in phloem-related activities than non-viruliferous whiteflies, although differences were not statistically significant (WDI phloem-related variables). No significant differences were observed in the total duration of probing time “Probe” nor in the duration of the intercellular stylet pathway “C” waveform per insect (WDI). However, the duration of the “Probe” event (waveform duration per event, WDE) was significantly longer in viruliferous than in non-viruliferous whiteflies (5.01±0.71 min *vs.* 3.58±0.35 min, respectively). Furthermore, significant differences were found in the duration of “C” per event (WDE), being shorter for viruliferous than for non-viruliferous whiteflies. Although there was no significant differences in the number (NWEI) and the duration of “pd” per insect (WDI) we observed that viruliferous *B. tabaci* made more intracellular punctures per insect (“pd”) (5.24±1.68) of higher duration (32.15±10.35 s) when compared with non- viruliferous whiteflies (NWEI: 2.50±0.77; WDI: 17.85±4.82 s). We found no differences on xylem ingestion values (“G”) between treatments for NWEI, WDI and WDE but there were significant differences between the number of viruliferous and non-viruliferous whiteflies (PPW = 9/17 *vs* 16/18 respectively) that performed xylem ingestion.

**Table 2:** Mean (± standard error) non-sequential EPG variable values (ranges in parenthesis) for the probing behavior of TYLCV-viruliferous and non-viruliferous *B. tabaci* adults on healthy eggplants during an eight-hour recording^a^.

Non-sequential variables	Whitefly	PPW	P^b^	NWEI	P^c^	WDI	P^c^	WDE	P^c^
**Non-probe**	Non-viruliferous	18/18	0.999	85.28±9.34 (19–168)	0.116^1^	176.62±21.74 (71–401)	0.487^1^	2.08±0.17 (2×10^−2^–107)	0.087^2^
	Viruliferous	17/17		65.12±12.06 (10–189)		155.44±20.09 (42–319)		2.39±0.30 (2×10^−2^–261)	
**Probe**	Non-viruliferous	18/18	0.999	84.83±9.31 (19–167)	0.118^1^	303.33±21.75 (79–409)	0.717^2^	3.58±0.35 (2×10^−2^–207)	0.000 ^2^
	Viruliferous	17/17		64.82±12.07 (10–189)		324.56±20.09 (161–438)		5.01±0.71 (2×10^−2^–313)	
**C**	Non-viruliferous	18/18	0.999	86.00±9.36 (21–168)	0.146^1^	218.87±20.25 (51–361)	0.115^1^	2.54±0.17 (2×10^−2^–108)	0.000 ^2^
	Viruliferous	17/17		67.41±12.05 (11–190)		170.63±21.92 (55–336)		2.53±0.31 (2×10^−2^–179)	
**pd** d	Non-viruliferous	11/18	0.555	2.50±0.77 (0–10)	0.257^2^	17.85±4.82 (3–46)	0.218^2^	4.36±0.32 (2–15)	0.599^1^
	Viruliferous	12/17		5.24±1.68 (0–26)		32.15±10.35 (4–141)		4.34±0.25 (1–12)	
**G**	Non-viruliferous	16/18	0.028	1.22±0.15 (0–2)	0.152^2^	59.45±10.03 (17–150)	0.821^2^	43.23±8.37 (3–143)	0.165^1^
	Viruliferous	9/17		1.59±0.64 (0–8)		94.16±34.08 (9–325)		31.39±7.54 (4–186)	
**E1**	Non-viruliferous	6/18	0.063	0.39±0.14 (0–2)	0.015 ^2^	1.24±0.47 (1×10^−1^–3)	0.087^1^	1.07±0.31 (1×10^−1^–2)	0.720^1^
	Viruliferous	11/17		1.71±0.54 (0–9)		5.16±1.99 (5×10^−1^–23)		1.96±0.66 (2×10^−1^–17)	
**E2**	Non-viruliferous	5/18	0.028	0.33±0.14 (0–2)	0.011 ^2^	97.54±37.36 (20–196)	0.177^1^	81.28±34.54 (1–196)	0.843^1^
	Viruliferous	11/17		1.24±0.29 (0–4)		157.92±23.18 (63–284)		82.72±15.37 (1–242)	
**sE2**	Non-viruliferous	5/18	0.028	0.28±0.11 (0–1)	0.009 ^2^			97.41±37.42 (20–196)	0.606^1^
	Viruliferous	11/17		1.00±0.21 (0–2)				101.34±15.84 (20–242)	

a **PPW**, proportion of individuals that produced the waveform type; **NWEI**, number of waveform events per insect; **WDI**, waveform duration (min) per insect; **WDE**, waveform duration (min) per event [52]. **Non-probe**, non-probe activity; **Probe**, probe activity. Waveforms: **C**, intercellular stylet pathway; **pd**, short intracellular punctures; **G**, xylem ingestion; **E** shows phloem-related activities: **E1**, correlates with salivation into phloem sieve elements [38]; **E2**, regards as ingestion from phloem that is comparable to E2 of aphids [49]; **sE2**: sustained E2 (longer than 10 minutes).

b P-values according to a Chi-square 2×2 goodness of fit test or by a Fisher exact test when the expected values were lower than 5. Underline-type indicates significant differences (p≤0.05).

c P-values according to a Student t-test^1^ for Gaussian distribution variables and Mann Whitney U-test^2^ for non-Gaussian distribution variables. Underline-type indicates significant differences (p≤0.05).

d Potential drop (pd) duration is expressed in seconds.

Table 3 shows sequential EPG variables that describe the sequence of events related to each other during the eight hours of recording. The total duration of E1 followed by sustained E2 (longer than 10 min) (WDI) was three times longer in viruliferous than in non-viruliferous whiteflies (p = 0.047). It is important to note that the longer is the duration of salivation into the phloem sieve elements (E1) the higher is the probability of transmission of TYLCV by *B. tabaci* [38]. The proportion of sustained E2 events respect to the total number of E2 events was not significantly different between non-viruliferous (90.0±10.0%) and viruliferous (87.9±6.4%) whiteflies (data not shown), but a higher significant number of viruliferous whiteflies (PPW = 11/17) were able to produce sustained phloem ingestions than non-viruliferous whiteflies (PPW = 5/18) (Table 2). Despite there were no significant differences in the “time elapsed from the first probe to the first E” and in the “time elapsed from first probe to the first sustained E2” the duration of both variables was shorter for viruliferous than for non-viruliferous *B. tabaci* whiteflies. No significant differences (p>0.05) were found in the variables “time to first probe from start of EPG”, “duration of first probe” and “duration of second probe”, indicating that pre-phloematic differences between TYLCV-viruliferous and non-viruliferous *B. tabaci* did not occur.

**Table 3:** Mean (± standard error) sequential EPG variable values (ranges in parenthesis) for the probing behavior of non- viruliferous and viruliferous *B. tabaci* on healthy eggplants during an eight-hour recording^a^.

Sequential variables	Whitefly	PPW	P^b^	NWEI	P^c^	WDI	P^c^
**Time to 1^st^ probe from start of EPG**	Non-viruliferous	15/18	0.172			4.34±1.06 (1×10^−1^–12)	0.372^2^
	Viruliferous	15/17				7.08±2.26 (7×10^−2^–32)	
**Duration of 1^st^ probe**	Non-viruliferous	18/18	0.999			1.48±0.51 (2×10^−2^–10)	0.086^2^
	Viruliferous	17/17				0.86±0.26 (4×10^−2^–5)	
**Duration of 2^nd^ probe**	Non-viruliferous	18/18	0.999			1.18±0.35 (3×10^−2^–5)	0.322^2^
	Viruliferous	17/17				19.22±18.37 (8×10^−2^–313)	
**Time from the beginning of the 1^st^ probe to 1^st^ pd**	Non-viruliferous	11/18	0.555			162.63±28.19 (38–312)	0,699^1^
	Viruliferous	12/17				145.53±27.51 (19–316)	
**Time from the beginning of that probe to 1^st^ E**	Non-viruliferous	6/18	0.063			11.40±2.45 (4–22)	0.132^2^
	Viruliferous	11/17				18.15±5.17 (7–69)	
**Time from 1^st^ probe to 1^st^ sustained E2 (10 min)**	Non-viruliferous	18/18	0.999			415.40±27.33 (108–480)	0.069^2^
	Viruliferous	17/17				298.41±40.75 (80–480)	
**Time from the beginning of that probe to 1^st^** **sustained E2 (10 min)**	Non-viruliferous	5/18	0.028			13.92±2.22 (11–23)	0.126^2^
	Viruliferous	11/17				21.34±5.13 (10–70)	
**Time from 1^st^ probe to 1^st^ E**	Non-viruliferous	18/18	0.999			395.94±29.07 (108–480)	0.106^2^
	Viruliferous	17/17				291.17±40.85 (79–480)	
**Number of probes to the 1^st^ E1**	Non-viruliferous	6/18	0.063	59.33±6.38 (34–77)	0.261^1^		
	Viruliferous	11/17		45.82±16.36 (2–185)			
**Number of probes after 1^st^ E**	Non-viruliferous	18/18	0.999	12.11±5.55 (0–80)	0.207^2^		
	Viruliferous	17/17		13.06±4.78 (0–66)			
**Number of probes (shorter than 3 minutes) after 1^st^ E**	Non-viruliferous	18/18	0.999	9.94±4.56 (0–67)	0.207^2^		
	Viruliferous	17/17		11.18±4.53 (0–66)			
**Total duration of E1 followed by E2**	Non-viruliferous	5/18	0.028			1.47±0.50 (5×10^−1^–3)	0.126^2^
	Viruliferous	11/17				4.93±1.91 (5×10^−1^–22)	
**Total duration of E1 followed by sustained E2** **(>10 min)**	Non-viruliferous	5/18	0.028			1.10±0.35 (5×10^−1^–2)	0.047 ^2^
	Viruliferous	11/17				3.10±0.81 (5×10^−1^–10)	

a **PPW**, proportion of individuals that produced the waveform type; **NWEI**, number of waveform events per insect; **WDI**, waveform duration (min) per insect [52]. **Non-probe**, non-probe activity; **Probe**, probe activity. Waveforms: **C**, intercellular stylet pathway; **pd**, short intracellular punctures; **G**, xylem ingestion; **E** shows phloem-related activities: **E1**, correlates with salivation into phloem sieve elements [38]; **E2**, regards as ingestion from phloem that is comparable to E2 of aphids [49]; **sE2**: sustained E2 (longer than 10 minutes).

b P-values according to a Chi-square 2×2 goodness of fit test or by a Fisher exact test when the expected values were lower than 5. Underline-type indicates significant differences (p≤0.05).

c P-values according to a Student t-test^1^ for Gaussian distribution variables and Mann Whitney U-test^2^ for non-Gaussian distribution variables. Underline-type indicates significant differences (p<0.05).

## Discussion

Most of the effects of plant virus on their vectors reported so far refer to plant-mediated indirect modifications that provoke changes in the behavior or performance of their vectors when alighting or feeding on diseased plants [20]–[23], [25], [53]. Our work, however, shows that virus-vector direct interactions may also take place, particularly on circulative-transmitted viruses that remain in the vector’s body for their entire lifespan. Our findings show that the begomovirus TYLCV can directly modify the settling and feeding behavior of its insect vector, *B. tabaci*. In this sense, an interesting recent study indicates that although begomovirus may cause a negative direct effect on its vector, decreasing the sugars:amino-acids ratio in honeydew excreted by viruliferous whiteflies, feeding by the vector on virus-infected plants may improve nutritional assimilation and consequently performance of the vector [25]. Other virus-vector direct interactions between plant viruses and their insect vectors are not neutral. TSWV tospovirus, the only plant member of the Bunyaviridae family, activates the immune system of its vector, the thrips *F. occidentalis* [54]. Similarly, the feeding behavior of TSWV-infected *F. occidentalis* males is modified in a way that likely enhances virus transmission [30]. Tospoviruses are transmitted in a propagative-circulative manner, which means they replicate in their vectors and are inoculated by the saliva during feeding. The animal infecting members of the Bunyaviridae family have the ability to produce direct effects on their vectors as well, with viral infections resulting in increased biting rates that enhances the virus transmission rate of infected mosquitoes [2]. These findings suggest that behavioral modification of vectors is a highly adaptive trait for plant- and animal-infecting parasites [30].

In the same way, geminiviruses may constitute a family of plant viruses that are in the process of acquiring or losing abilities to interact actively with their insect vector *B. tabaci*, to a point reminiscent of a host-pathogen relationship [17]. Ghanim et al. [43] indicated that TYLCV is transmitted to the progeny of viruliferous insects (transovarial transmission). Furthermore, the results obtained by Rubinstein and Czonek [32] showed that the passage of TYLCV in its vector, *B. tabaci* is not neutral. TYLCV DNA is retained in the insect during its entire adult life, the virus significantly shortens the lifespan and has a negative effect on vector fecundity. Also, the virus can be sexually transmitted from insect to insect [55]. In our work, the focus was to compare if settling, probing and feeding behavior of *B. tabaci* was altered after acquisition of TYLCV. Our settling behavior assays show that the acquisition of TYLCV by *B. tabaci* promotes a decrease in velocity and duration of the movement that results in a reduction of the distance moved once whiteflies alight on their host plant. Furthermore, the delay in the start of movement by viruliferous whiteflies might mean that virus affects insect behavior in two different ways. On one respect, the virus may affect negatively the insect locomotive system slowing down its movement (arrestant behavior); on the other hand, the virus may provoke whiteflies to focus their attention on feeding activities as soon as they land and settle on the plant. The probing and feeding behavior results obtained in our work using the EPG technique suggest that in fact, TYLCV-viruliferous *B. tabaci* is more efficient in regard to feeding efficiency than non-viruliferous whiteflies. Viruliferous insects reached their feeding target site -phloem sieve elements- more often and were more likely to attain sustained phloem ingestion than non-viruliferous whiteflies (Table 2).

Phloem feeding by its vector *B. tabaci* is an absolute prerequisite for transmission of begomoviruses such as TYLCV. These viruses are inoculated during salivation into phloem sieve elements (E1) just before any phloem sap ingestion (E2) begins. TYLCV as many other plant viruses relies heavily on its vector’s ability to move it to a new host. Our results show behavioral modifications in viruliferous-whiteflies that are predictive of increased virus passage from plant to plant. We observed that viruliferous *B. tabaci* spent less time on the intercellular stylet pathway phase and longer time on phloem-associated activities, increasing the number of salivation and ingestion events into the phloem sieve elements (E1 and E2). Virions of TYLCV are transmitted from the salivary gland of *B. tabaci* to the phloem sieve elements during the salivation phase; consequently, the significant higher total duration of E1 followed by sustained E2 events observed in TYLCV-viruliferous whiteflies can be associated to a higher transmission efficiency. In fact, Jiang et al. [38] revealed that the duration of the salivation phase into the phloem sieve elements was the most significant variable associated with the inoculation of TYLCV by *B. tabaci*. Furthermore, the same work shows that the number and duration of E1 was strongly and positively correlated with the transmission efficiency of TYLCV by *B. tabaci*. A similar conclusion was reported for BYDV, which is transmitted by the aphid *Rhopalosiphum padi* during the E1 phase [49]. In our study, viruliferous whiteflies reached the phloem sooner, and made longer and higher number of E1 salivation events, increasing the chances and the efficiency of TYLCV transmission.

Our study demonstrates that when *B. tabaci* females acquire TYLCV, there is an obvious change in their behavior leading to enhanced transmission efficiency and spread of the virus. Conversely, the vector may also benefit from such interaction providing that its feeding efficiency -ability to find the phloem- was increased after virus acquisition, suggesting that TYLCV-*B. tabaci* interaction results in a mutualistic relationship.

The modification of vector behavior trait adds a selective advantage by increasing virus dissemination, a similar situation to that reported for TSWV-*F. occidentalis* interactions [30]. In the latter study males but not females modified its feeding habits after acquisition of TSWV leading to a higher number of superficial non-ingesting probes that are known to be associated with virus transmission. In the case of TSWV, infected males are more efficient vectors than infected females because of their specific feeding habits that avoid cell damage. In our study, however, we focused on female whiteflies because they are known to be 5 times more efficient than males in transmitting TYLCV [56]. Further studies should be conducted to find out if the settling or feeding behavior of TYLCV-viruliferous *B. tabaci* males is modified or not.

It is reasonable here to point out some limitations of our experimental design that aimed to concentrate exclusively on the direct effects of TYLCV on its insect vector *B. tabaci* by using eggplant, which is a non-host plant for the virus. The procedure used cannot totally discard some plant-mediated indirect effects resulting from previous experience of whiteflies when feeding on virus-infected tomato plants during the acquisition access period. The exposure of whiteflies to TYLCV-infected and non-infected tomato plants might influence their subsequent behavior on the eggplant leaf discs or plants. Nevertheless, whiteflies were starved for 30 min and 1 h prior to the settling and feeding behavior experiments, respectively, which very likely minimized the risk of any possible host-plant mediated effects on whitefly behavior. Further research should be conducted to clarify if the behavior of viruliferous *B. tabaci* on a virus-immune plant may be influenced by previous feeding experience on a TYLCV-infected plant. The evolutionary implications of our findings are that behavioral modification of vectors may be a conserved trait among different families and members of plant and animal viruses. In fact, a recent study, reported that the acquisition of an aphid-transmitted luteovirus directly manipulates the behavior of its aphid vector to maximize transmission [31]. In their work they showed that non-infective aphid vectors are attracted to virus-infected host plants, which is beneficial as it increases the chances for virus acquisition. After the virus is acquired, the vector preferences shift to non-infected hosts, maximizing pathogen transmission potential. On this sense, further research needs to be done with viruliferous *B. tabaci* feeding on infected and non-infected tomato host plants to study if TYLCV alters the host selection behavior of its whitefly vector.

Our work and other recent findings [24], [26] suggest that the transmission mechanisms shape pathogens effects on host-vector interactions. We conclude that TYLCV, a circulative phloem-restricted begomovirus, has evolved to manipulate the settling, probing and feeding behavior of its vector, *B. tabaci* in a manner that facilitates its own transmission. This knowledge has clear implications for understanding the epidemiology of insect-transmitted plant diseases and improving their management options under integrated agricultural systems.

## Supporting Information

Video S1 Fast speed valid video recording (13x) of *Bemisia tabaci* displacement on an eggplant leaf disc. Valid videos were those in which the whiteflies remained within the experimental arena during the 10-min recording time.
(MP4)

Video S2 Fast speed invalid video recording (13x) of *Bemisia tabaci* displacement on an eggplant leaf disc. Invalid videos were those in which whiteflies started to fly or abandoned the experimental arena during the 10-min recording time.
(MP4)
